# Up‐regulation of mir‐10b predicate advanced clinicopathological features and liver metastasis in colorectal cancer

**DOI:** 10.1002/cam4.789

**Published:** 2016-09-04

**Authors:** Hong Jiang, Jijun Liu, Yingtao Chen, Chong Ma, Baosong Li, Tao Hao

**Affiliations:** ^1^ Department of General SurgeryBinzhou Medical University HospitalBinzhou256603China

**Keywords:** Clinicopathological, colorectal cancer, CRC, Kaplan–Meier survival curve, liver metastasis, microRNA‐10b

## Abstract

Given the emerging role of microRNA in tumor disease progression, we investigated the association between miRNA 10b expression, liver metastasis, and clinicopathological of colorectal cancer (CRC). Two hundred and forty‐six pairs of samples (including CRC samples and normal adjacent tissues) from CRC patients were collected from May 2004 to May 2009. All samples verified to contain at least 80% tumor cells, and were immediately frozen in liquid nitrogen and stored at −80°C or fixed in 10% formalin for paraffin embedding. The expression of miRNA‐10b in CRC tissues was evaluated using a quantitative real‐time polymerase chain reaction RT‐PCR. Correlation between miR‐10b expression and poor clinicopathological of CRC patients were analyzed using Student's *t*‐tests and Chi‐square tests. A Kaplan–Meier survival curve was generated following a log‐rank test. miR‐10b expression was up‐regulated in CRC tissues (P < 0.0001) and in patients diagnosed as colorectal liver metastasis (CLM) at initial involvement or during follow‐up. When the Tumor Node Metastasis (TNM) stage was taken into consideration, the expression levels of miR‐10b were positively correlated with advanced TNM stages. In addition, the miR‐10b expression of patients diagnosed as CLM at initial involvement was significantly higher than those without liver metastasis (nCLM). Similarly, those patients developed with CLM during follow‐up (FCLM) was also markedly higher than those with nCLM. miR‐10b expression was also found correlated with advanced stage (*P* < 0.0001), lymph node metastasis (*P* = 0.025), venous infiltration (*P* = 0.007), poorer differentiation (*P* = 0.002), and served as an independent prognostic factor of poor overall survival (*P* < 0.0001). This study demonstrated the expression of miR‐10b had strong potential to serve as a noninvasive biomarker for CRC prognosis and predicting liver metastasis.

## Introduction

Colorectal cancer (CRC) is a major cause of cancer death worldwide, with over 1.2 million new cases diagnosed each year along with over 600,000 deaths per year 1. According to GLOBOCAN statistics for Romania 2008, age‐standardized rate for CRC ranked fourth in incidence and third in mortality among all tumor types 1. Majority of the patients could have relatively good outcomes with 5‐year survival ranging from 50% to 90% 2 depending upon the initial stage of the disease and other prognostic factors. However, although the treatment of CRC definitely improved during the past decades, the 5‐year survival rate for patients with metastatic CRC remains poor. Liver metastasis, which is the most common site for metastatic spread of CRC, is observed in 20–25% of patients at initial diagnosis, and eventually develops after resection of the primary CRC in a further 40–50% of patients 3. Radical liver resection remains the only potentially curative therapy for patients with CLM, with reported 5‐ and 10‐year actuarial survival rates of 17–35% and 16–23%, respectively 4. However, most CRC patients with liver metastasis are not candidates for surgical treatment, with the 5‐year survival rate below 10% 5. Although the prognosis of patients with CLM has been improved by recent advances in multidisciplinary treatments, liver metastasis is still one of the major determinants of survival 6. Therefore, it is urgently necessary to unravel the underlying molecular mechanisms and genetic alterations that lead to CRC metastases.

Recently, it was reported that miRNA alteration and dysfunction play critical roles during tumorigenesis and metastasis by way of the regulation of cancer cell proliferation, differentiation, apoptosis, and invasion 7, 8, 9. MicroRNAs (miRNAs) belong to a conserved group of short, endogenous, and noncoding RNAs (18–24 nucleotides) that regulate the expression of a wide variety of genes. Through base pairing with the 30‐untranslated region (30‐UTR) of target genes, miRNAs enhance mRNA degradation or inhibit posttranscriptional translation 10. For instance, miR‐140‐5p inhibits tumor growth and metastasis in hepatocellular carcinoma by targeting TGFBR1 and FGF9 11; down‐regulation of miR‐224 and the passenger strand of miR‐221 increase MBD2, suppressing maspin and promoting colorectal tumor growth and metastasis in mice 12; miR‐137 suppresses CRC invasion and metastasis by way of regulating FMNL2 13; miR‐26a plays a role in tumorigenesis and metastasis and have implications to develop new strategies for cancer therapy 14. Although miRNAs have been extensively investigated in recent years, the molecular regulatory mechanisms of miRNAs and their significance in CLM remain largely unknown and need exploring. In particular, previous studies demonstrated that CRC invasion and metastasis were stimulated by miR‐21 and miR‐103/107 by down‐regulating tumor suppressors PDCD4, and DAPK and KLF4, respectively, but inhibited by miR‐137 and miR‐30a by down‐regulating oncogenes FMNL2 and PIK3CD, respectively 13, 15, 16, 17.

miRNA‐10b (miR‐10b) has been reported to play a role in the invasion and metastasis of cancer 18. miR‐10b was initially found highly expressed in metastatic breast cancer cell lines, able to generate metastases when growing as a primary tumor in mice 19. miR‐10b is also found to be a tumor enhancer in non‐small cell lung cancer (NSCLC), which may represent a potential therapeutic target for NSCLC intervention 20. Moreover, another study showed that miR‐10b expression is correlated with survival and was a predictor of the poor outcome in patients with esophageal squamous cell carcinoma 21. Recently, using a large dataset of CRC miRNA and gene expression profiles, miRNA‐10b was found significantly differentially expressed between primary colorectal carcinoma and liver metastases 22. In this study, we aimed to explore the association between the expression of miR‐10b in CRC and clinicopathological features, and to evaluate its value in prognosis of this tumor.

## Materials and Methods

### Samples and cases

Two hundred and forty‐six pairs of samples (including CRC samples and normal adjacent tissues) from CRC patients were collected from May 2004 to May 2009 at Binzhou Medical University Hospital. None of the patients received chemotherapy or radiotherapy before the surgery. The clinicopathological variables such as gender, age, tumor stage, histological style were analyzed. All samples verified to contain at least 80% tumor cells, and were immediately frozen in liquid nitrogen and stored at −80°C or fixed in 10% formalin for paraffin embedding. Cases with familial adenomatous polyposis CRC were excluded from the study. The miR‐10b quantitative analysis was performed with those samples via real‐time polymerase chain reaction (PCR). The study was approved by the Ethics Committee of Binzhou Medical University Hospital. Written informed consent was obtained for the acquisition and use of patient tissue samples and anonymized clinical data. The diagnosis and histological grade of each case were confirmed by two pathologists independently and based on the clinicopathological criteria described by the UICC.

### RNA extraction and qRT‐PCR

For real‐time PCR analysis of miRNA, the total RNA was extracted from the paraffin embedded tissue samples using the RecoverAll^™^ Total Nucleic Acid Isolation Kit (Applied Biosystems, Foster City, CA) according to the manufacturer's instructions. The concentration and purity of the RNA were determined using UV spectrophotometry (A260/A280 ratio of 1.8–2.0). Denaturing agarose gel electrophoresis was also performed to visually assess the RNA, which contained highly abundant 28S and 18S ribosomal RNA bands. Quantitative PCR (qRT‐PCR) was performed using the miScript PCR System (Qiagen, Hilden, Germany) according to the manufacturer's instructions. The relative expression was calculated using the comparative cycle threshold (CT) method and normalized to the expression of U6 small nuclear (sn) RNA. The changes in the expression were calculated by the 2 ^−∆∆CT^ method using the equation, relative quantity = 2 ^−∆∆CT^, where ∆∆CT = (CT ^miR−21^–CT ^U6^)_cancer_‐ (CT ^miR−21^–CT ^U6^) _normal adjacent tissues_, and CT is the cycle threshold for each specimen.

### Statistical analyses

To analyze baseline characteristics, the continuous variables were presented as Mean ± SD and compared between groups by the Student's *t*‐tests, and the categorical data were compared by Chi‐square tests. Associations between miR‐10b expression and over survival of the patients with CRC were estimated using adjusted relative risks and 95% confidence intervals (95% CIs) from multivariate logistic regression. Survival time was calculated from the date of CRC diagnosis to the date of death or last follow‐up. Survival analysis was estimated using the Kaplan–Meier method, log‐rank test, and Cox proportional hazards regression model. The *P* < 0.05 was considered to indicate a statistically significant difference. As previous study found the expression of miR‐10b measured in liver metastasis showed a statistically significant association with the survival of patients affected with stage IV CRC (hazard ratio = 1.47) 22, the sample size was calculated to be at least 200 each group with a power of 80% and a *P*‐value of 0.05 using a statistical programme (PASS 11). The software of SPSS version 13.0 for Windows (SPSS, Inc., Chicago, IL) was used for statistical analysis.

## Results

### miR‐10b expression in CRC tissues

**Figure 1:**
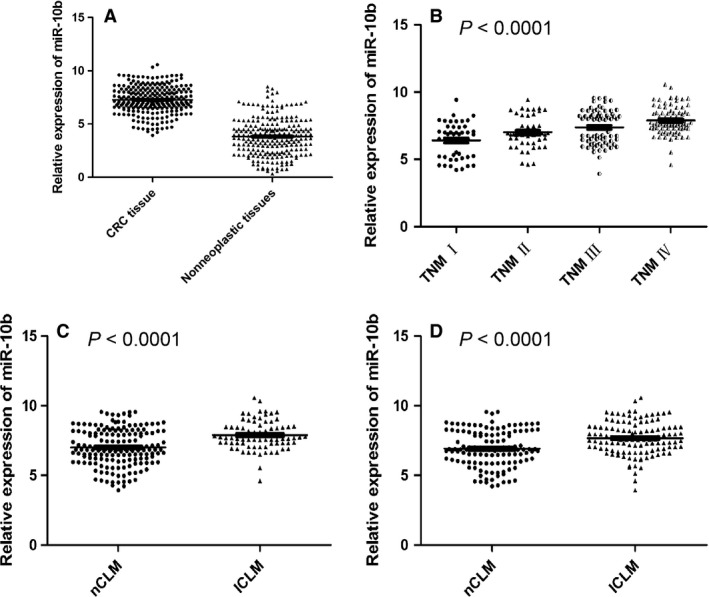
miR‐10b expression in colorectal cancer (CRC) and adjacent nonneoplastic tissues detected by quantitative real‐time polymerase chain reaction (qRT‐PCR) analysis.

miR‐10b expression was detected in 246 pairs of CRC tissues and adjacent nonneoplastic tissues normalized to RNU6B. As shown in Figure 1, we found that the expression of miR‐10b was markedly increased in CRC tissues compared with nonneoplastic liver tissues (mean ± SD: 7.267 ± 1.286 vs. 3.815 ± 1.692, *P* < 0.0001, Figure 1A). When the TNM stage was taken into consideration, the expression levels of miR‐10b were positively correlated with advanced TNM stages (*P* < 0.0001, Figure 1B). In addition, the miR‐10b expression of patients diagnosed as CLM at initial involvement (ICLM) was significantly higher than those without liver metastasis (nCLM) (Figure 1C). Similarly, those patients developed with CLM during follow‐up (FCLM) was also markedly higher than those with nCLM (Figure 1D).

### Expression of miR‐10b and clinicopathological features

We then analyzed the association between miR‐10b expression and clinicopathological parameters. CRC tissues expressing miR‐10b at levels less than the median expression level (7.29) were assigned to the low expression group (mean expression value 6.23, *n* = 123), and those samples with expression above the median value were assigned to the high expression group (mean expression value 8.30, *n* = 123). The high level of miR‐10b expression was significantly more common in CRC tissues with advanced pathologic grade than those with low pathologic grade (*P* < 0.0001, Table 1). Besides, miR‐10b up‐regulation group has a higher rate of venous infiltration (*P* = 0.007) and lymph node metastasis (*P* = 0.025). No significant association was found between miR‐10b expression and gender, age, or histological type at diagnosis. When the tumor differentiation was taken into consideration, the higher expression of miR‐10b was found in the patients with poor differentiation than in patients with well differentiation (*P* = 0.002).

**Table 1:** Correlation of miR‐10b expression with clinicopathological features of colorectal cancer (CRC) (*n* = 246)

Clinicopathological features		No. of cases	miR‐10b expression		*P*
			Low	High	
Age (year)	<60	88	46	42	0.690
	≥60	158	77	81	
Gender	Male	176	86	90	0.672
	Female	70	37	33	
TNM stage	I	47	34	13	<0.0001
	II	48	36	12	
	III	73	35	38	
	IV	78	18	60	
Tumor Stage	Early stage (I‐II)	95	70	25	<0.0001
	Advanced stage (III‐IV)	151	53	98	
Histological type	Adenocarcinoma	186	89	97	0.299
	Mucinous adenocarcinoma	60	34	26	
Lymph node metastasis	Positive	173	78	95	0.025
	Negative	73	45	28	
Venous invasion	Positive	114	46	68	0.007
	Negative	132	77	55	
Differentiation	Poor	119	47	72	0.002
	Well	127	76	51	

**Table 2:** Multivariate analyses of different prognostic parameters in patients with colorectal cancer (CRC) by Cox regression analysis

Parameter	Risk ratio	95% CI	*P*
Age	1.32	0.52–2.37	0.680
Gender	1.25	0.36–3.18	0.796
Histological type	3.58	1.74–6.16	<0.001
Lymph node metastasis	1.94	1.27–4.18	<0.001
Venous infiltration	2.55	1.45–5.61	<0.001
TNM stage	1.85	1.16–3.64	<0.001
Differentiation	5.11	3.68–8.72	<0.0001
miR‐10b expression	4.63	2.67‐7.54	<0.0001

CI, Confidence interval.

### miR‐10b expression and survival in patients with CRC

**Figure 2:**
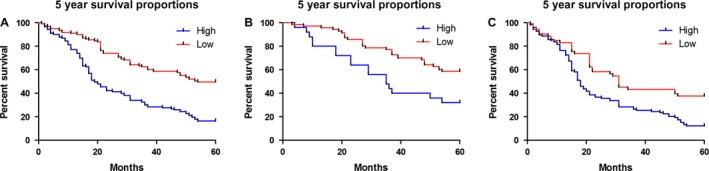
Kaplan–Meier survival curves for colorectal cancer (CRC) patients with high or low expression of miR‐10b. (A) The 5‐year overall survival rate of all CRC patients with high or low miR‐10b expression; (B) The 5‐year overall survival rate of CRC patients with early stage in high or low miR‐10b expression group; (C) The 5‐year overall survival rate of CRC patients with advanced stage in high or low miR‐10b expression group.

In the 5 years' follow‐up, the association between miR‐10b expression and prognosis was detected using Kaplan–Meier method and log‐rank test. The overall survival of CRC patients with high miR‐10b expression was significantly shorter than that with low miR‐10b expression (*P* < 0.0001, Figure 2A). When the pathological stages were considered, the higher miR‐10b expression was a risk of poor prognosis in both the early stage of (*P* = 0.006, Figure 2B) and the advanced CRC (*P* = 0.0006, Figure 2C).

### Multivariate Cox proportional hazard analysis

In the Cox proportional hazard model, it was confirmed that miR‐10b expression in the biopsy samples (RR 4.63; 95% CI, 2.67–7.54), differentiation (RR 5.11; 95% CI, 3.68–8.72), tumor stage (RR 1.85; 95% CI, 1.16–3.64), venous infiltration (RR, 2.55; 95% CI, 1.45–5.61), lymph node metastasis (RR 1.94; 95% CI, 1.27–4.18), and histological type (RR 3.58; 95% CI, 1.74–6.16) were predictor of poor prognosis of the patients with CRC. Our results showed that age and gender were not independent predictor of the survival of patients with HCC.

## Discussion

This study investigates the potential clinical utility of miR‐10b to serve as noninvasive prognostic and liver metastasis‐predictive biomarkers in patients with CRC. We observed that miR‐10b expression was up‐regulated in CRC tissues compared with noncancerous tissues. In addition, the miR‐10b expression of patients diagnosed as CLM at initial involvement or during follow‐up was also found markedly higher than those with no liver metastasis. Moreover, the up‐regulation of miR‐10b in CRC cancer tissues was also significantly correlated with aggressive clinicopathological features. We found that the patients with high miR‐10b expression have an advanced tumor staging, higher risk of lymph node metastasis and venous infiltration, and poorer differentiation. The results of Kaplan–Meier analyses showed that CRC patients with the high miR‐10b expression tend to have shorter overall survival and progression‐free survival. The multivariate analysis clearly indicated that the high miR‐10b expression in biopsy samples may be considered as an independent prognostic factor in CRC for decreased survival.

Despite achievements in the treatment in the few past decades, CRC remains a major public health concern, resulting in more than 600,000 deaths each year. The major cause of death and relapse from CRC is metastasis, colorectal tumors often metastasize to the liver and the extent of liver infiltration is a major determinant of survival 23. Given this, understanding the molecular mechanisms that promote CLM is of crucial significance to the development of therapeutic strategies for CRC patients. However, we have a relatively poor understanding of metastases though significant progress has been made characterizing the molecular features of primary tumors. Studies have found that genetic alterations in tumor cells lead to cellular heterogeneity, which might promote cancer cell invasiveness and colonization in specific organs during the metastatic process. Sequencing reveals that liver metastases may be genetically distinct from primary CRC tumors 24. However, tumors readily adapt to new microenvironments and many of these changes are reflected in changes in microRNA expression 25. It has also been revealed that more than 50% of miRNAs are located at tumor‐related genomic regions or in fragile sites 26. Additionally, a considerable advantage of implementing miRNAs as novel molecular tools derives from the fact that a single miRNA can target and regulate the expression of hundreds of mRNAs. Therefore, it is easier to work with a small number of miRNAs than with mRNAs to discover biomarkers of interest with higher sensitivity and specificity.

The relationship between colorectal liver metastasis and miRNA has been reported. Chen et al. reported that high expression of miR‐103 and miR‐107 was associated with the liver metastasis potential of CRC cell lines and poor prognosis in patients with CRC 16. Siemens et al. showed that miR‐34a is preferentially down‐regulated via hypermethylation in primary colon cancers, which were associated with distant metastases, whereas miR‐34b/c seems to be silenced irrespective of tumor stage 27. Hur et al. showed that miR‐200c plays an important role in mediating epithelial–mesenchymal transition and metastatic behavior from CRC to liver 28. Overexpression of miR‐122 and concomitant suppression of CAT1 in primary tumors appears to play important roles in the development of colorectal liver metastasis 29. In addition, it was reported in the analysis of clinical samples using quantitative real‐time RT‐PCR that miR‐21, ‐22, and ‐143 participate in colorectal liver metastasis 30, 31. The association between miR‐10b was rarely researched in CRC, but elevated miR‐10b expression and poor prognosis were reported in several other tumor types like malignant glioma, pancreatic cancer, and nasopharyngeal carcinoma 32, 33, 34, 35, 36, 37, 38, 39. miR‐10b was previously proposed to be positively correlated with metastatic potential of breast cancer cells 19. Another study by the same group reported reduced pulmonary metastases by silencing of miR‐10b 40. MicroRNA‐10b overexpression promotes non‐small cell lung cancer cell proliferation and invasion 20. More recently, increased miR‐10b levels have been associated with bone metastases as well as spread to the lymph nodes 41, 42.

The mechanisms of tumor metastasis and invasive functions of miR‐10b appear to be tissue specific and depend upon the expression pattern of its target mRNAs and gene targets in a given cell type. miR‐10b was reported to have a role in regulating angiogenesis in gliomagenesis 35, 43. Overexpression of miR‐10b leading to cancer metastasis has also been correlated with the metastasis‐promoting transcription factor Twist which induces epithelial‐to‐mesenchymal transition (EMT) 19. miR‐10b requires Twist to induce EMT and the resulting cell motility and invasiveness in the breast epithelial cells. E‐cadherin, another important determinant of EMT has also been proposed to be a target of miR‐10b 44. In addition, miR‐10b has a prominent role in regulating tumor invasion and metastasis by targeting the HOXD10, a transcription factor known for its roles in cellular migration and extracellular modeling such as RhoC, uPAR, *α*3‐integrin, and MT1‐MMP 19, 45, 46, 47, 48, 49. Silencing of miR‐10b significantly increases the levels of HOXD10 to inhibit metastasis 50. miR‐10b expression has also been shown to correlate with the migration and invasion of human esophageal cancer cell lines through regulation of Kruppel‐like factor 4 (KLF4) expression 51. Additionally, the reported downstream targets for miR‐10b also include T‐lymphoma invasion and metastasis‐1 factor 52, stress‐induced cell surface molecule MICB 53, tat‐interacting protein 30 34, etc.

In conclusion, our results have demonstrated that the levels of miR‐10b are higher in CRC tissues than those in matched normal tissues and correlated with disease stage, the presence of lymph node metastasis and venous infiltration, and tumor differentiation. More importantly, the miR‐10b expression of patients diagnosed as CLM at initial involvement or during follow‐up was found markedly higher than those with no liver metastasis. These findings enhance our understanding of the role of miR‐10b in CRC progression and liver metastasis and suggest that miR‐10b may function as microtumor promoter genes in CRC. These findings suggest the potential clinical use of microRNA measurements, particularly in estimating prognosis for patients with CRC. Large well‐designed studies with diverse populations and functional evaluations are warranted to confirm and extend our findings. Examining new targets and other biological experiments will clarify the functions and roles of microRNAs in CLM.

## Conflict of Interest

None declared.
